# Sex Hormones and Abdominal Muscle Area and Radiodensity in Men: The Multi-Ethnic Study of Atherosclerosis

**DOI:** 10.21203/rs.3.rs-3909259/v1

**Published:** 2024-02-12

**Authors:** Amar Osmancevic, Matthew Allison, Iva Miljkovic, Chantal A Vella, Pamela Ouyang, Penelope Trimpou, Bledar Daka

**Affiliations:** University of Gothenburg; UC San Diego; University of Pittsburgh; University of Idaho; Johns Hopkins School of Medicine; University of Gothenburg and Sahlgrenska University Hospital; University of Gothenburg

## Abstract

Information on the associations of testosterone levels with abdominal muscle volume and quality in men is limited, while the role of estradiol and SHBG on these muscle characteristics are unclear. To investigate the association between fasting serum sex hormones and CT-derived abdominal muscle area and radiodensity in adult men. Cross sectional observational study using data from the Multi-Ethnic Study of Atherosclerosis. A community-based sample of 907 men aged 45–84 years; 878 men with complete data were included in the analysis. CT scans of the abdomen were interrogated for muscle characteristics. Multivariable linear regressions were used to test the associations. After adjustment, higher levels of both total testosterone and estradiol were associated with higher abdominal muscle area (1.79, 0.1–3.4, & 1.79, 0.4–3.2, respectively). In the final analyses, levels of total testosterone showed a positive association, while an inverse relationship was observed for SHBG with abdominal muscle radiodensity (0.3, 0.0–0.6, & −0.34, −0.6 - −0.1, respectively). Our results indicate a complex association between sex hormones and abdominal muscle characteristics in men. Specifically, total testosterone and estradiol were associated with abdominal muscle area, while only total testosterone was associated with muscle radiodensity and SHBG was inversely associated with muscle radiodensity.

## INTRODUCTION

Abdominal obesity is linked to a higher risk of cardiometabolic disorders and mortality.^1^ However, recent evidence suggests the quality and quantity of abdominal muscles may also play an important role in cardiometabolic health.^2^ In this regard, abdominal muscle radiodensity, measured in Hounsfield Units (HU), reflects the quality of muscle tissue and the degree of fat infiltration/fibrosis.^3^ Of note, growing evidence suggests that abdominal muscle radiodensity is inversely associated with cardiovascular events and mortality in men.^2^

Earlier studies showed testosterone induces muscle fiber hypertrophy and increases the number of myonuclear cells by regulation of protein synthesis, satellite cells and stem cell proliferation, in addition to stimulation of the myogenic lineage and inhibition of the adipogenic cell line by activating androgen receptors (ARs).^4,5^ Moreover, in men with testosterone deficiency, replacement therapy improves muscle volume, strength and quality of life.^6^ However, it is unknown whether testosterone has different effects on different abdominal muscle functional groups. In addition, the effects of estradiol on muscle characteristics in men are unclear. In this regard, Russel and Colleagues reported in their review, no clear effect of estradiol on muscle mass or strength in men.^7^ Yet, other studies have shown that estradiol could play a key role in regulating abdominal adiposity but is also directly associated with the volume of lean mass in men and appears to prevent expansion of adiposity.^,7-9^

While there is some evidence to suggest that both testosterone and estradiol play an important role in the regulation of muscle function and volume, the nature of this relationship is not yet well established. Given this, we examined the cross-sectional associations between sex hormones and abdominal muscle characteristics in a large multi-ethnic sample of middle-aged and older men and hypothesized that higher estradiol and testosterone levels would be associated with greater abdominal muscle area, while testosterone would also be associated with higher abdominal muscle radiodensity.

## RESULTS

Baseline characteristics of the study population are presented in Table 1. The mean age was 61.6 years. The majority of participants where non-Hispanic White (42 %), followed by Hispanic/Latino (27 %), African American (17 %) and Chinese American (14 %). On average, men were overweight with a mean BMI of 27.6 kg/m^2^. The participants reported an average of 12 hours a week of physical activity. Moreover, 42% of participants were hypertensive, 13 % stated active cigarette smoking, 15 % had diabetes mellitus, and 24% were taking a cholesterol-lowering medication. The mean *total testosterone* level was 15 nmol/L.

### Association between Sex Hormones and Abdominal Muscle Area

*Total* testosterone was significantly associated with total abdominal muscle area in the first model (B=1.39, 95 % CI 0.0 - 2.8, p = 0.05), which was accentuated with further adjustment (Model 2: 1.81, 0.2 - 3.5, p = 0.03; Model 3: 1.79, 0.1 - 3.4 p < 0.01) (Table 2). No significant associations were found between *total testosterone* and abdominal *stabilizing* muscle area (Table 3), while the associations were significant in all models for abdominal *locomotor* muscle area (Table 4).

Levels of estradiol were significantly associated with total abdominal muscle area in all three models: Model 1 (2.14, 0.8 - 3.6, p < 0.01), Model 2 (1.97, 0.6 - 3.4, p < 0.01), Model 3 (1.79, 0.4 - 3.2, p = 0.01) with similar results for abdominal locomotor and abdominal stabilizing muscle area (Table 3 and Table 4).

No significant associations were found between levels of *free* testosterone and total abdominal muscle areas (Table 2), as well as stabilizing. However, significant associations were presented between free testosterone and locomotor area in model 1 & model 2(0.38, 0.0 – 0.7, p = 0.04, 0.37, 0.0 – 0.7, p = 0.04) with borderline significance in model 3 (0.37, −0.0, 0.7, p = 0.05), respectively (Table 2, 3 & 4).

### Associations between Sex Hormones and Abdominal Muscle Radiodensities

*Total* testosterone was significantly associated with total abdominal muscle radiodensity in Models 2 and 3, but not in Model 1 (Model 1: 0.04, −0.2 - 0.3, p = 0.79; Model 2; 0.32, 0.1 - 0.7, p = 0.04; Model 3: 0.3, 0.0 - 0.6, p = 0.04) (Table2). Similar results were found for radiodensity of stabilizing muscles (Table 3), but not for locomotor muscle (Table 4).

No significant associations were found between *free* testosterone and abdominal muscle radiodensities in fully adjusted models (Table 2, 3 & 4).

No significant associations were found between levels of estradiol and total abdominal and stabilizing muscle radiodensity (Table2 & 3), but there was a borderline significant association between estradiol and abdominal locomotor muscle radiodensity ((Model 1: −0.27, −0.0 - 0.6, p = 0.09: Model 2: 0.28, −0.0 - 0.6, p = 0.07; Model 3: 0.26, −0.0 - 0.6, p = 0.09) (Table 4).

Higher SHBG levels were associated with a lower radiodensity of abdominal muscle in all models (Model 1: −0.35, −0.6 - −0.1, p = 0.02: Model 2: −0.35, −0.6 - −0.1, p = 0.02; Model 3: −0.34, −0.6 - −0.1, p = 0.02) (Table 2). The results were similar for abdominal stabilizing and locomotor muscles (Table 3).

### Associations between Sex Hormones and Abdominal Muscle Area Indexes

A significant association was found in all models for *total* testosterone and TAMAi (Model 1: B= 0.10, 0,0 - 0.2, p < 0.01, Model 2: 0.11, 0.1 - 0.2, p < 0.01, Model 3: 0.10, 0.1 - 0.2, p <0.01) (Table 2). That is, in fully adjusted models, one SD increase in testosterone levels resulted with an increase of 0.10 cm^2^/(weight/height^2^) in abdominal muscle area index. Similar relationships were observed between *total* testosterone and abdominal locomotor and stabilizing muscle area indices (Table 3 & 4).

Estradiol was found to be significantly associated with total abdominal muscle index (TAMAi) in model 1 (B = 0.10, 0.0 - 0.1, p= 0.03), which was borderline significant in Models 2 and 3 (Model 2: 0.05, −0.0 - 0.1, p= 0.06, Model 3: 0.05, −0.0 - 0.1, p= 0.06). Significant associations were found for estradiol and abdominal locomotor muscle area index in all three models but not for abdominal stabilizing muscle area index (Table 3 and 4).

Significant associations were shown for free testosterone with total, stabilizing and abdominal muscle area index in fully adjusted models (0.08, 0.0, 0.1, p = 0.008, 0.05, 0.0 – 0.1, p = 0.03, 0.02, 0.0 - 0.04, p = 0.02), respectively. Inverse non-significant associations were found between levels of SHBG and abdominal muscle areas, and muscle area indexes in both model 2 and model 3.

## DISCUSSION

Our study presents novel findings on the associations between sex hormones and SHBG and abdominal muscles in men. First, our results indicate that increases in serum levels of total testosterone and estradiol were associated with significant increases in abdominal muscle mass in men. In fact, our data indicate that the associations were stronger for estradiol than total testosterone with abdominal muscle mass to include both stabilizing and locomotor muscles. Second, significant associations were found between higher levels of total testosterone and abdominal muscle radiodensities. Third, our study presented a significant negative association between SHBG and abdominal muscle radiodensity.

A significant association was found between *total* testosterone and total abdominal muscle area, with similar associations presented for abdominal locomotor muscle area but not for abdominal stabilizing muscle area. A plausible explanation of these differences might be that locomotor muscle contains a greater number of types II myofibers, a more dynamic and power related muscle type.^10^ Type I myofibrils, which are predominantly found in the abdominal stabilizing muscles, have shown to be rather associated with endurance and higher lipid content.^10-12^ Even though some studies have shown that supraphysiologic levels of testosterone increase both type I and II myofibers equally, other studies have reported testosterone affects maximal voluntary strength rather than endurance.^5,13^

Free testosterone was positively associated with total abdominal muscle area and radiodensity although, significance was found for locomotor muscle area in model 1 and 2 with borderline significance in model 3. *Total* testosterone was positively associated with TAMAi, abdominal stabilizing and locomotor muscle area indexes. In concurrence with our findings, Han et al. reported similar outcomes presented between *total* testosterone and abdominal muscle area index in men.^14^ However, no adjustments were made for SHBG.

In our study, *total* testosterone was associated with increased total and stabilizing abdominal muscle radiodensities, independent of confounding factors.

Our results suggest that *total* testosterone is significantly associated with the degree of radiodensity of abdominal muscles, including muscle size. *Total* testosterone mainly includes SHBG-bound testosterone which has long been assumed to be inactive. However, recent experimental studies have shown the endocytic Megalin receptor, found in human skeletal myocytes, transports SHBG-bound testosterone and estradiol into cells. ^15,16^ This could indicate *total testosterone* may have an active role in cell regulation and muscle activity.

Our study found that higher levels of estradiol were significantly associated with higher levels of all abdominal muscle areas. Estradiol has earlier been found to play a key role in regulation of myokines, i.e., skeletal muscle proteins, with critical functions associated with exercise-related benefits and inflammation regulation in tissues.^17^ Similarly, in a study on elderly Swedish men, estradiol, and not testosterone, was associated with lean mass measured with DEXA.^8^

Estradiol was positively but non-significantly associated with total and stabilizing muscle radiodensities, while a borderline-significant association was shown with locomotor muscle radiodensity. This would be in concurrence with a study by Wiik et al., reporting higher concentrations of estradiol receptors in skeletal muscles of men engaged in greater endurance training.^18^ Although the role of estradiol on skeletal muscle characteristics is unclear, one study in men reported that supplementation of estradiol increased lipid utilization in skeletal muscles, increasing strength.^19^

The bioactive role of SHBG is still debated. An inverse association has been shown between SHBG levels and insulin resistance and metabolic syndrome. ^48^ Other studies have found a positive association between SHBG and inflammatory cytokines, low protein diet and hip fractures in elderly even after adjustment for sex hormones.^20,21^ Our study showed an inverse association between SHBG and all abdominal muscle densities. Other results have reported an inverse relationship between SHBG and lean muscles measured by DEXA.^8^ Furthermore, Yuki et al. reported SHBG levels were significantly higher in the group of individuals diagnosed with sarcopenia compared to the normal group.^22^ In agreement with our findings, SHBG has been reported to have a significant inverse association with muscle strength in elderly men.^23^ One plausible cause of the negative associations between SHBG and abdominal muscle radiodensities could be that an increase in SHBG concentrations may influence the binding capacity and magnitude of available free testosterone and could suggest a partial explanation to some of the weaker association found for other sex hormones .^24^

This study has a number of strengths, including usage of data from a large and diverse cohort, detailed sampling of information with validated instruments as well as standardized sampling of blood specimens according to guidelines.^25^ Furthermore, by assessing muscle composition with CT, we were able to estimate its quality. However, our study does have some limitations. First, radioimmunoassay technique (RIA) was used to measure sex hormones and SHBG. This has been described to be less precise than mass spectrometry in the measurement of sex-hormone levels.^26^ Furthermore, levels of sex hormones may be affected by the presence of several cross-reacting steroids.^27^ Second, *free* testosterone was calculated and not directly measured which has been shown to overestimate levels compared with laboratory measured free testosterone.^28^ While the Sodergard method has previously been described as one of the most common methods for calculating free testosterone in endocrinology literature, it has limitations, including higher estimates compared to other algorithms and accurate mainly when competing steroids to binding sites are limited and normal levels of SHBG are involved.^29^ Furthermore, the Sodergard method presents concordant results to the Vermuelen algorithm and its association constant was validated when compared to results of calculations with a gold standard technique.^29^ Third, the sex hormones were measured at visit 1 whilst CT scans were made at visit 2 and visit 3. We partially addressed this limitation by adjusting for the time from baseline to CT scan as a confounder in model 2 and model 3. In addition, measurements of physical activity and sedentary behavior were self-reported.

We only evaluated abdominal muscle area and radiodensity, and therefore, our findings may not be applicable to peripheral muscles. Finally, this study design was observational and cross-sectional, which is prone to residual confounding, as well as temporal and selection biases.

## CONCLUSION

In this analysis, we demonstrate a positive association between total testosterone levels with abdominal muscle area and radiodensity, whereas estradiol showed a similar strong association with abdominal muscle area but not radiodensity. Additionally, SHBG was significantly and inversely associated with abdominal radiodensity although a negative trend was presented for abdominal muscle index. These results suggest the relevance of sex hormone levels to maintain muscle mass and density with advancing age.

## MATERIAL AND METHODS

### Study Design and Study Population

At baseline (2000 to 2002), 6814 adult men and women between 45–84 years that were free of clinical cardiovascular disease were recruited into the Multi-Ethnic Study of Atherosclerosis (MESA). Participants were enrolled from six US communities (New York [NY], Baltimore [MD], Chicago [IL], Los Angeles [CA], Twin Cities [MI] and Winston-Salem [NC]). Approximately 38% were Non-Hispanic White, 28% African American, 23% Hispanic American, and 11% Chinese American.

### Data Collection

Details on the MESA cohort methods have been published.^30^ Briefly, trained staff performed specimen blood draws and processing of venous blood samples, blood pressure measurements and all interviews. Using standard procedures, fasting blood samples were processed and stored at −80°C.^31^

Information on lifestyle factors, medications and co-morbidities were gathered using validated questionnaires. Race/ethnicity was self-reported at baseline according to 2000 US Census criteria. All individuals treated with sex hormones at baseline were excluded from the study. Physical activity (hours/week) and sedentary behavior (hours/week) were measured by using a comprehensive, semiquantitative questionnaire.^32^ Current medication use was assessed according to standardized questionnaires. ^33^ Hypertension was defined as a systolic blood pressure above 140 mmHg and/or a diastolic above 90 mmHg or taking a blood pressure lowering medication. Diabetes mellitus was defined as self-reported diabetes or use of glucose lowering medications. ^34,35^ Measurement of high-sensitivity C-reactive protein (hsCRP), a marker of systemic inflammation, has previously been described. ^31,36^

### Computed Tomography for Body Composition

A random subset of 1970 participants (946 men) at visits 2 and 3 (2002 to 2005) were enrolled in an ancillary study obtaining abdominal computed tomography (CT) scans, which were then interrogated for abdominal muscle area, abdominal radiodensity, visceral adipose tissue and subcutaneous fat tissue. At three clinical sites (Northwestern University, University of California Los Angeles, and Johns Hopkins University) electron-beam CT scanner (Imatron C-150) was used while at the remaining clinical sites (Columbia University, Wake Forest University, and University of Minnesota) multi-detector CT scanners (Sensation 64 GE lightspeed, Siemens S4 Volume Zoom, and Siemens Sensation 16) were used. CT scans were set at a collimation of 3mm with a slice thickness of 6 mm. In total, six cross-sectional slices were taken at L2/L3, L3/L4 and L4/L5 intervertebral disc spaces. Approximately half of the participants underwent CT scans at visit two and the other half at visit three.

Assessment of abdominal muscles and adipose tissue were obtained from these CT scans and have been described earlier.^37^ Using a semi-automated method, measurement of total tissue, lean muscle, and adipose tissue were assessed using Medical Imaging Processing Analysis and Visualization (MIPAV) software version 4.1.2 (National Institutes of Health, Bethesda, Maryland). Abdominal tissue was categorized by Hounsfield units (HU) with – 190 to −30 HU assessed as adipose tissue, −30 to −0 HU defined as mixed connective tissue, while values 0 to 100 HU were set as lean muscle. ^38,39^ Abdominal muscle area and adipose tissue area were calculated by summing the number of pixels while muscle radiodensity was defined by average HU value measured within that muscle’s corresponding fascial plane. Research staff responsible for analyzing CT scans were blinded to participants’ clinical information. The inter- and intra-rater reliability of measurements for total abdominal area as well as measurements for all muscle groups was 0.99 and 0.93 to 0.98, respectively.^28^

Visceral adipose tissue was determined as fat tissue in the visceral cavity, excluding intermuscular fat. Four abdominal muscle groups were assessed bilaterally. Area and radiodensity of the obliques, rectus abdominus and paraspinalis muscle groups comprised the muscles of stabilization, while the psoas muscles were the locomotor group. Combined area and radiodensity of muscles of stabilization and locomotion were defined as total abdominal muscle area (TAMA) and radiodensity (TAMD), respectively.

Adjustment for body mass index (BMI, kg/m^2^) was made for abdominal muscle areas and were defined as abdominal muscle indexes (total abdominal muscle area index (TAMAi) (TAMAi = TAMA/BMI), stabilizing muscle area index (TSMA/BMI) and locomotor muscle area index (TLMA/BMI).^40^

**Figure 1:**
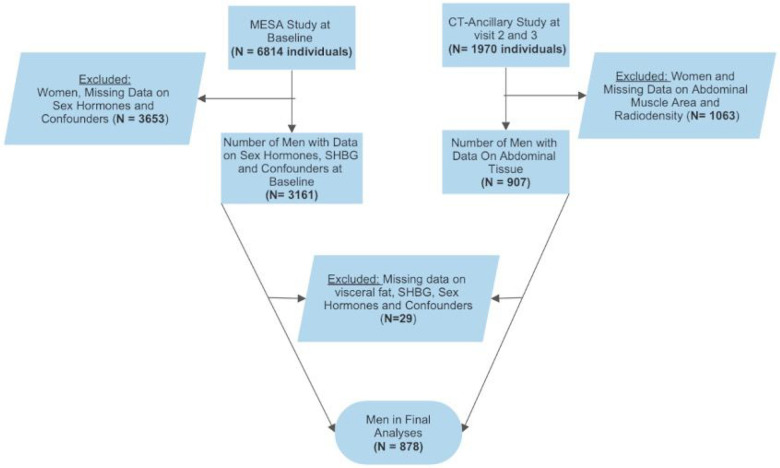
Flow-chart showing the number of men included in the final analyses.

For this analysis we excluded participants with missing data from CT scans, anthropometric measurements, endogenous sex hormones, lifestyle factors, co-morbidities, and medication use. The final sample size included 878 men (Fig. 1).

#### Assessment of endogenous sex hormones

Measurement of endogenous sex hormone levels have previously been described. ^41,42^ In brief, total testosterone was measured using radioimmunoassay kits. Sex hormone binding globulin (SHBG) concentration was assessed by Chemiluminescent enzyme immunometric assay (Immulite kits, Diagnostic Products Corporation, Los Angeles, CA). A ~ 10% blind pool was obtained to assess quality control serum. The coefficients of variation for total testosterone and SHBG were 12.3% and 9.0%, respectively. ^43^ Estradiol was measured using an ultra-sensitive radioimmunoassay kit (Diagnostic System Laboratories, Webster, TX) with a coefficient of variation of 10.5%. ^43^ Free testosterone( nmol/L) was calculated according to the method of Södergård. ^44^

### Statistical Analysis

Continuous variables were reported as means and standard deviations (SD) while categorical variables were shown as frequencies and percentages. Abdominal muscle areas/radiodensities were the outcome variables. Total, locomotor and stabilizing abdominal muscles showed normal distributions. As such, multivariable linear regression models were conducted to assess the associations between levels of sex hormones and abdominal muscles. The outcome was defined as the change in HU for abdominal muscle radiodensity and square centimeters for abdominal muscle area for each 1-SD increment change in levels of testosterone, estradiol and SHBG.

Model 1 adjusted for age, race/ethnicity, and visceral adipose tissue. Model 2 included variables from model 1 and SHBG (when investigating the associations of total testosterone and estradiol), CRP physical activity, sedentary behavior, cigarette smoking, alcohol use, and time from baseline to CT. Model 3 included variables from Model 2 with further adjustment for hypertension, diabetes mellitus, dyslipidemia, use of cholesterol/thyroid/hypertension medication. No adjustment was made for SHBG when free testosterone was the independent variable.

A two-tailed *p*-value < 0.05 was considered as statistically significant. Analyses were conducted using IBM SPSS Statistics, version 29.

### Ethical Considerations

The MESA study protocol was approved by the Institutional Review Board at the Johns Hopkins University Hospital, University of California Los Angeles, University of Minnesota, Wake Forest University Hospital, Northwestern University Hospital, and Columbia University. All methods were performed in accordance with the relevant guidelines and regulations as set by the approving institutions in a standardized manner. Written informed consent was given by all participants ^45^.

## Figures and Tables

**Table 1.** Baseline characteristics of the study population.

MEN (N= 878)	Mean ± SD/N (%)
Age (years)	61.6 (± 10.02)
SBP (mmHg)	125.7 (± 19.3)
DBP (mmHg)	75.4 (± 9.3)
Total Abdominal Muscle Area (cm^2^)	116.3 (± 24.1)
Total Abdominal Muscle Area Index (cm^2^/BMI)	4.26 (± 5.0)
Total Abdominal Muscle Radiodensity (HU)	44.4 (± 5.0)
Abdominal Stabilizing Muscle Area (cm^2^)	87.4 (± 20.2)
Abdominal Stabilizing Muscle Radiodensity (HU)	42.1 (± 5.4)
Abdominal Locomotor Muscle Area (cm^2^)	29.1 (± 6.1)
Abdominal Locomotor Muscle Radiodensity (HU)	51.3 (± 5.0)
Abdominal Visceral Fat Area (cm^2^)	163.0 (± 70.9)
Abdominal Subcutaneous Fat Area (cm^2^)	209.0 (± 92.7)
BMI (kg/m^2^)	27.6 (± 4.2)
WHR	0.96 (± 0.1)
hsCRP (mg/L)	2.5 (± 4.35)
TT (nmol/L)	15.0 (± 5.6)
fT (pmol/L)	0.3 (± 0.1)
SHBG (nmol/L)	43.4 (±18.2)
Estradiol (nmol/L)	0.12 (± 0.0)

Race/Ethnicity	
Non-Hispanic White	368 (42 %)
Chinese American	125 (14 %)
Black	153 (17%)
Hispanic/Latino	229 (27 %)

Time from baseline to CT (years)	2.63 (± 1.0)
Total Physical activity (hours/week)	11.99 (± 5.5)
Sedentary Behavior (hours/week)	24,8 (± 14.5)
Current cigarette smoker	115 (13 %)
Diabetes Mellitus	129 (15 %)
Hypertension	368 (42%)
Cholesterol medicine use	213 (24%)

SBP (systolic blood pressure), DBP (diastolic blood pressure), BMI (body mass index), WHR (waist-hip-ratio), hsCRP (high sensitivity c-reactive protein), SHBG (sex-hormone binding globulin), fT (free Testosterone), TT (Total Testosterone)

**Table 2 (a-c).** Association between levels of serum testosterone (total and free), SHBG, estradiol and abdominal muscle variables.

2a) Total Abdominal Muscle Area							
Testosterone		fT		SHBG		ESTRADIOL	
B	95% CI	B	95% CI	B	95% CI	B	95% CI
MODEL 1							
1.39	−0.0, 2.8	1.09	−0.4, 2.5	0.24	−1.3, 1.8	2.14	0.8,3.6
model 2							
1.81	0.2, 3.5	1.11	−0.3, 2.6	0.24	−1.3, 1.8	1.97	0.6, 3.4
MODEL 3							
1.79	0.1, 3.4	1.14	−0.3, 2.6	0.21	−1.3, 1.8	1.79	0.4, 3.2
2b) Total Abdominal Muscle Area INDEX							
Testosterone		fT		SHBG		ESTRADIOL	
B	95% CI	B	95% CI	B	95% CI	B	95% CI
MODEL 1							
0.1	0.0, 0.2	0.1	−0., 0.1	0.0	0.0, 0.1	0.10	0.0, 0.1
MODEL 2							
0.1	0.1, 0.2	0.1	0.0, 0.1	0.0	0.0, 0.1	0.05	0.0, 0.1
MODEL 3							
0.1	0.1, 0.2	0.1	0.0, 0.1	0.0	0.0, 0.1	0.05	0.0, 0.1
2C) Total Abdominal Muscle RADIOdENSITY							
Testosterone		fT		SHBG		ESTRADIOL	
B	95% CI	B	95% CI	B	95% CI	B	95% CI
MODEL 1							
0.04	−0.2, 0.3	0.16	−0.1, 0.4	−0.35	−0.6, −0.1	0.17	−0.1, 0.4
MODEL 2							
0.32	0.1, 0.7	0.19	−0.1, 0.5	−0.35	−0.6, −0.1	0.16	−0.1, 0.4
MODEL 3							
0.32	0.1, 0.6	0.21	−0.1, 0.5	−0.35	−0.6, −0.1	0.16	−0.1, 0.4

Linear regressions are used to investigate the associations in three models. Model 1(adjustment for age, race/ethnicity, and visceral adipose tissue), model 2(adjustment for model 1 + SHBG, CRP, physical activity, sedentary behavior, cigarette smoking, alcohol use and time from baseline to CT), model 3 (adjustment for model 2 + hypertension, diabetes mellitus, dyslipidemia, cholesterol medication, thyroid agents), SHBG (sex-hormone binding globulin), CRP (C-reactive protein), fT (free testosterone).

**Table 3(a-c).** Association between testosterone (total and free), SHBG, estradiol and abdominal stabilizing muscles.

3a) Abdominal STABILIZING Muscle Area							
Testosterone		fT		SHBG		ESTRADIOL	
B	95% CI	B	95% CI	B	95% CI	B	95% CI
MODEL 1							
0.87	−0.35, 2.1	0.71	−0.5, 1.95	0.07	−1.2, 1.38	1.45	0.2, 2.6
model 2							
1.16	−0.3, 2.6	0.74	−0.5, 2.0	0.06	−1.3, 1.4	1.30	0.1, 2.5
MODEL 3							
1.16	−0.3, 2.6	0.77	−0.5, 2.0	0.07	−1.3, 1.4	1.22	0.0, 2.4
3B) Abdominal Stabilizing Muscle Area INDEX							
Testosterone		fT		SHBG		ESTRADIOL	
B	95% CI	B	95% CI	B	95% CI	B	95% CI
MODEL 1							
0.10	0.0, 0.1	0.0	−0.0, 0.1	0.3	0.0, 0.1	0.04	0.0, 0.1
model 2							
0.07	0.0, 0.1	0.0	0.0, 0.1	0.03	0.0, 0.1	0.03	0.0, 0.10
MODEL 3							
0.10	0.0, 0.1	0.1	0.0, 0.1	0.02	0.0, 0.1	0.03	0.0, 0.10
3C) Abdominal Stabilizing Muscle RADIODENSITY							
Testosterone		fT		SHBG		ESTRADIOL	
B	95% CI	B	95% CI	B	95% CI	B	95% CI
MODEL 1							
0.08	−0.2, 0.4	0.20	−0.1, 0.5	−0.31	−0.6, 0.0	0.14	−0.2, 0.4
model 2							
0.36	0.2, 0.7	0.23	−0.1, 0.5	−0.31	−0.6, 0.0	0.11	−0.2, 0.4
MODEL 3							
0.35	0.1, 0.7	0.3	−0.0, 0.6	−0.29	−0.6, 0.0	0.11	−0.2, 0.4

Linear regressions are used to investigate the associations in three models. Model 1(adjustment for age, race/ethnicity, and visceral adipose tissue), model 2(adjustment for model 1 + SHBG, CRP, physical activity, sedentary behavior, cigarette smoking, alcohol use and time from baseline to CT), model 3 (adjustment for model 2 + hypertension, diabetes mellitus, dyslipidemia, cholesterol medication, thyroid agents), SHBG (sex-hormone binding globulin), CRP (C-reactive protein), fT (free testosterone).

**Table 4 (a-c).** Association between testosterone (total and free), SHBG, estradiol and abdominal locomotor muscles.

4a) Abdominal LOCOMOTOR Muscle Area							
Testosterone		fT		SHBG		ESTRADIOL	
B	95% CI	B	95% CI	B	95% CI	B	95% CI
MODEL 1							
0.52	0.2, 0.9	0.4	0.0, 0.7	0.17	−0.2, 0.6	0.7	0.3, 1.1
model 2							
0.65	0.2, 1.1	0.4	0.0, 0.7	0.18	−0.2, 0.6	0.67	0.3, 1.0
MODEL 3							
0.63	0.2, 1.0	0.4	−0.0, 0.7	0.14	−0.3, 0.5	0.65	0.3, 1.0
4B) Abdominal LOCOMOTOR Muscle Area INDEX							
Testosterone		fT		SHBG		ESTRADIOL	
B	95% CI	B	95% CI	B	95% CI	B	95% CI
MODEL 1							
0.05	0.0, 0.1	0.0	0.0, 0.0	0.02	0.0, 0.0	0.02	0.0, 0.1
model 2							
0.10	0.0, 0.1	0.0	0.0, 0.0	0.02	0.0, 0.0	0.02	0.0, 0.1
MODEL 3							
0.10	0.0, 0.1	0.0	0.0, 0.0	0.01	−0.0, 0.0	0.02	0.0, 0.1
4C) Abdominal LOCOMOTOR Muscle RADIODENSITY							
Testosterone		fT		SHBG		ESTRADIOL	
B	95% CI	B	95% CI	B	95% CI	B	95% CI
MODEL 1							
−0.1	−0.4, 0.2	0.52	0.2, 0.9	−0.5	−0.8, −0.2	0.27	−0.0, 0.6
model 2							
0.23	−0.1, 0.6	0.1	−0.3, 0.4	−0.5	−0.8, −0.2	0.28	−0.0, 0.6
MODEL 3							
0.21	−0.2, 0.6	0.1	−0.2, 0.4	−0.5	−0.8, −0.2	0.26	−0.0, 0.6

Linear regressions are used to investigate the associations in three models. Model 1(adjustment for age, race/ethnicity, and visceral adipose tissue), model 2(adjustment for model 1 + SHBG, CRP, physical activity, sedentary behavior, cigarette smoking, alcohol use and time from baseline to CT), model 3 (adjustment for model 2 + hypertension, diabetes mellitus, dyslipidemia, cholesterol medication, thyroid agents), SHBG (sex-hormone binding globulin), CRP (C-reactive protein), fT (free testosterone).

## Data Availability

The supporting data for the conclusions drawn in this study can be obtained from the MESA committee, but access is subject to certain restrictions as they were utilized under license for the present study and are not publicly accessible. Nevertheless, interested parties can request access to the data directly from the authors, pending approval from the MESA committee. Amar Osmancevic retained unrestricted access to all data and takes accountability for both integrity of the data and the accuracy of the data analysis.
